# The path of pre-ribosomes through the nuclear pore complex revealed by electron tomography

**DOI:** 10.1038/s41467-019-08342-7

**Published:** 2019-01-30

**Authors:** Franck Delavoie, Vanessa Soldan, Dana Rinaldi, Jean-Yves Dauxois, Pierre-Emmanuel Gleizes

**Affiliations:** 1 LBME, Centre de Biologie Intégrative, Université de Toulouse, CNRS, UPS, 31062 Toulouse, France; 2 METi, Centre de Biologie Intégrative, Université de Toulouse, CNRS, UPS, 31062 Toulouse, France; 3 Institut de Mathématiques de Toulouse, UMR 5219, Université de Toulouse, CNRS, INSA, 31077 Toulouse, France

## Abstract

Determining the path of single ribonucleoprotein (RNP) particles through the 100 nm-wide nuclear pore complex (NPC) by fluorescence microscopy remains challenging due to resolution limitation and RNP labeling constraints. By using high-pressure freezing and electron tomography, here we captured snapshots of the translocation of native RNP particles through NPCs in yeast and analyzed their trajectory at nanometer-scale resolution. Morphological and functional analyses indicate that these particles mostly correspond to pre-ribosomes. They are detected in 5–6% of the NPCs, with no apparent bias for NPCs adjacent to the nucleolus. Their path closely follows the central axis of the NPC through the nuclear and inner rings, but diverges at the cytoplasmic ring, suggesting interactions with the cytoplasmic nucleoporins. By applying a probabilistic queueing model to our data, we estimated that the dwell time of pre-ribosomes in the yeast NPC is ~90 ms. These data reveal distinct steps of pre-ribosome translocation through the NPC.

## Abstract (web summary)

Large protein complexes and ribonucleoprotein particles (RNPs) such as pre-ribosomes are transported from the nucleus to the cytoplasm through the nuclear pore complex (NPC). Here the authors use ultrafast freezing and electron tomography to catch snapshots of native RNPs crossing the NPC and estimate their transit time using a probabilistic model.

## Introduction

The nuclear pore complex (NPC) forms a large channel through the nuclear envelope and mediates the exchange of macromolecules between the nucleus and the cytoplasm. It functions as a selective translocation barrier for the nucleocytoplasmic transport of a large variety of cargoes, from proteins and protein complexes to ribonucleoprotein (RNP) particles, including messenger (m)RNPs and pre-ribosomes^1,2^. The nuclear transport receptors (importins and exportins), that specifically bind the nuclear transport signals on the cargoes, overcome this translocation barrier by interacting with the nucleoporins (Nups), the components of the NPC. The structure of the NPC was determined at medium resolution in several species through integrative approaches^3^. A core domain, called the inner ring domain, lines the pore in the nuclear envelope. This inner ring is flanked by two outer rings: one on the cytoplasmic side, from which some Nups project into the cytosol, and the other on the nucleoplasmic side, which anchors a basket-like structure. The translocation barrier in the NPC comprises Nups that contain intrinsically disordered domains rich in phenylalanine and glycine (FG) repeats and occupy the central channel of the NPC. These FG-rich domains can assemble into a meshwork in vitro through interactions between their hydrophobic FG repeats. Such a meshwork was proposed to form a sieve-like hydrogel in the central channel, thereby creating the permeability barrier of the NPC^4^. Alternatively, although not exclusively, some FG-containing Nups (FG-Nups) may act as polymer brushes and filter the entry into the pore by entropic exclusion^5^. The precise organization of the central channel has eluded so far structural studies, but a dense structure called the central transporter was observed by electron microscopy^6,7^. The detailed structure of the NPC, together with indirect biochemical evidence, have also led some authors to propose that in addition to the central conduit, the NPC may also funnel cargoes through secondary peripheral channels^8,9^.

Direct observation of the path followed by native RNP particles through the NPC has been precluded by the difficulty to detect such complexes in the NPC at nanometer scale. Our current view of mRNP transport through the NPC largely relies on the observation of the giant Balbiani ring mRNPs^10,11^. Textbook images show how passage through the center of the NPC requires the complete remodeling of these mRNPs, whose unusually large size (~50 nm) exceeds by far the width of the NPC central channel, currently estimated to ~25–30 nm. However, this model may not apply to most mRNPs or to pre-ribosomal particles, whose size remains below this threshold^12^. More recently, fluorescence-tagged mRNPs were tracked by live microscopy in various cell systems to define both the trajectory and the dwell time of these cargoes in the NPC^13,14^. While these publications have revealed the trajectories of mRNPs upstream and downstream of the NPC, the spatial resolution of fluorescence microscopy studies in live cells remains too limited to determine the path of these cargoes within the NPC. In addition, to observe mRNPs by these techniques has only been possible so far with artificial mRNAs containing large arrays of hairpin tags (MS2 or PP7 sequences) in their 3′-UTR to recruit multiple fluorescent proteins, which may influence their behavior^13,14^.

Here, we report the direct observation of native RNP particles translocating through the NPC in yeast at nanometer resolution by means of electron tomography. We identify these particles as pre-ribosomes, analyze their distribution in the NPCs, and propose a model for their trajectory during translocation. In addition, by applying a probabilistic queueing model, we determine the time taken by these particles to traverse the NPC.

## Results

### Electron tomography reveals RNP particles in the NPCs

**Fig. 1:**
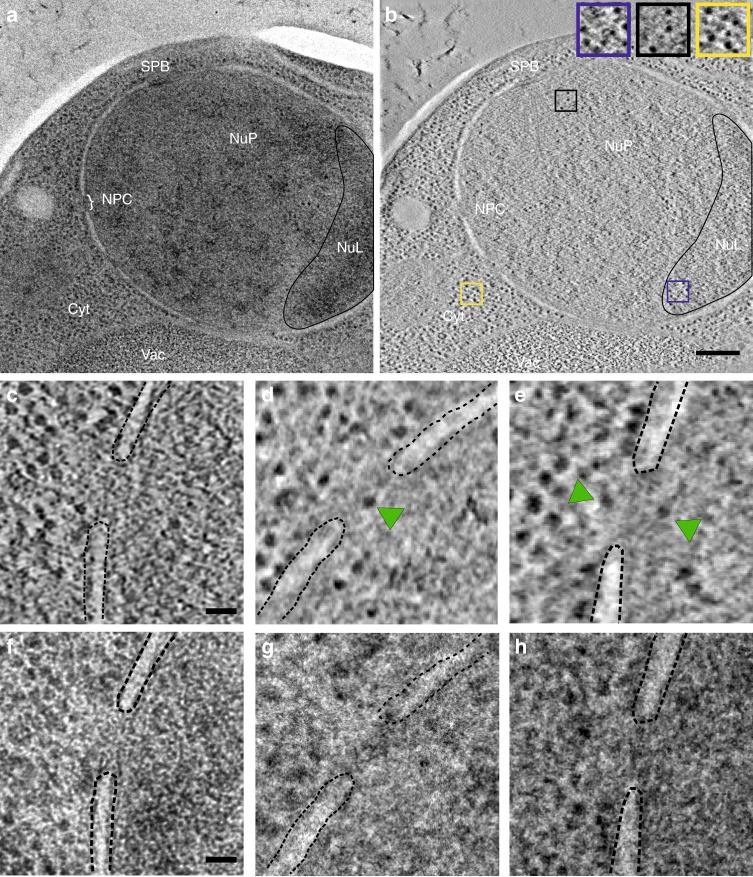
Electron tomography reveals large cargoes in NPCs. **a** High-pressure frozen yeast cell visualized by conventional transmission electron microscopy. Vac: vacuole, Cyt: cytoplasm, NuP: nucleoplasm, NuL: nucleolus (black line), SPB spindle pole body and NPC nuclear pore complex. Scale bar: 200 nm. **b** A section of the electron tomogram showing the same area as in (**a**). Nuclear electron-dense particles are detected in the crescent-shaped nucleolus (blue frame) and the nucleoplasm (black frame), while ribosomes are visible in the cytoplasm (yellow frame). Scale bar: 200 nm. **c**–**e** A selection of electron tomography images showing NPCs containing one or two large particles (green arrowheads). **f**–**g** These particles are not seen by conventional transmission electron microscopy. The dashed lines delineate the nuclear envelope. Scale bar: 50 nm

Previous estimates of the time taken by RNP particles to cross the NPC were between 10 and several hundreds of milliseconds^14^. To observe such transient events by electron microscopy, we froze cells of the yeast *Saccharomyces cerevisiae* wild-type strain NOY505 by high-pressure freezing, which brings the samples to sub-zero temperatures in <10 ms^15^. The frozen cells were then embedded in resin by freeze substitution, and electron tomography was performed on 80-nm ultrathin sections. Cell sections were chosen so that the nuclear envelope was cut perpendicularly, i.e. displayed clearly defined outer and inner nuclear membranes (Fig. 1a). In the resulting tomograms, numerous electron-dense particles resembling ribosomes (seen as black dots in the cytoplasm) were visible in the nucleolus as well as in the nucleoplasm (Fig. 1b). Pores in the nuclear membrane formed by NPCs were clearly identified but, in most cases, they contained no obvious cargo (Fig. 1c). However, within these pores or in their close vicinity (at a distance of ±40 nm max from each side of the NPC median plane), we could distinguish large particles apparently in transit or interacting with the NPC (Fig. 1d, e). These particles were seen by electron tomography (Fig. 1c–e) but not by conventional transmission electron microscopy (Fig. 1f–h). We systematically observed 566 NPCs and found large particles in 5.3% of them (Table 1). Of these 30 NPCs, 83% (*n* = 25) contained one particle (as in Fig. 1d), whereas 17% (*n* = 5) had two particles lining up in the pore (Fig. 1e). To test the reproducibility of this NPC occupancy rate, we performed electron tomography on strain OGP103, a strain of similar genetic background as NOY505. After observing 252 NPCs, we found the same proportion of NPCs occupied by a particle (5.6%, *n* = 14), including two NPCs containing two particles (Table 1). We pooled the results from the two strains to increase their statistical significance. We counted that 34% of the 818 counted NPCs contacted the nucleolus, while 66% were adjacent to the nucleoplasm. The 44 NPCs containing one or two particles followed the same distribution: 36% were in contact with the nucleolus and 64% in contact with the nucleoplasm. This indicates that nucleocytoplasmic translocation of these large cargoes is uniformly performed by NPCs in the nuclear envelope, irrespective of their position relative to the nucleolus or the nucleoplasm.

**Table 1:** Quantification of NPCs containing large, electron-dense particles

Strain	NOY505	OGP103	Total
Total NPCs	566	252	818
NPCs containing particles	30	14	44
Fraction of total NPCs (%)	5.3	5.6	5.4
NPCs containing 1 particle	25	12	37
NPCs containing 2 particles	5	2	7
Total number of particles in NPCs	35	16	51
Average number of particles/100 NPCs	6.2	6.3	6.2

### The RNP particles observed in NPCs are mostly pre-ribosomes

**Fig. 2:**
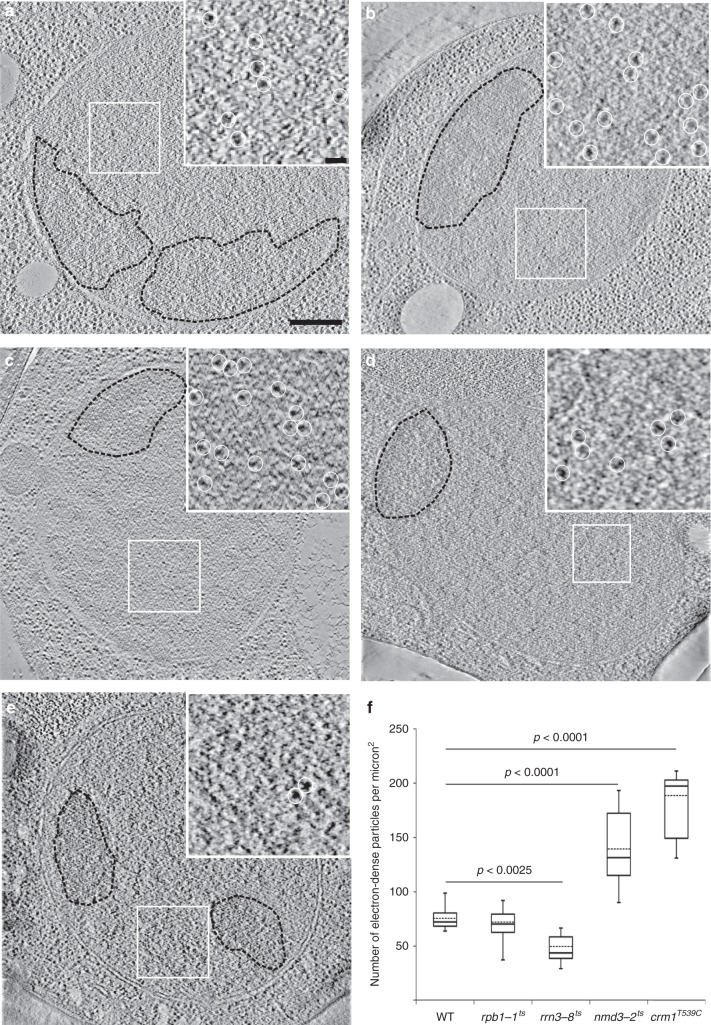
Identification of the particles in NPCs as pre-ribosomes. Tomographic sections of high-pressure frozen and freeze-substituted yeast strains: (**a**) NOY505 (wild-type), (**b**) MNY8 (*crm1*^*T539C*^*)* treated with LMB, (**c**) *nmd3-2*^*ts*^ at 37 °C, (**d**) *rpb1-1*^ts^ at 37 °C, and (**e**) *rrn3-8*^*ts*^ at 37 °C. The inset in each image shows an enlargement of the region of the nucleoplasm delineated by the white frame. The nucleolus is outlined by a dotted line and is better seen on the conventional TEM images of these areas (not shown). Electron-dense particles similar to those detected in NPCs (white circles) are observed in the nucleoplasm of wild-type cells. They strongly accumulate upon inactivation of Nmd3 and Crm1, and their number drops upon inactivation of RNA polymerase I transcription (*rrn3* mutant), which strongly supports that they correspond to pre-ribosomes. Along the same line, the levels of these particles are not affected by inactivation of RNA polymerase II (*rpb1* mutant). Scale bar: 500 nm. **f** Quantification of the density of nucleoplasmic electron-dense particles in the mutant strains compared to wild-type NOY505 cells. The box plot chart shows the median (line within the box), the mean (dash line within the box), and quartiles. Whiskers caps correspond to the maximum and minimum values. Statistical significance of the results was assessed by applying a one-way ANOVA test. Source data are provided as a Source Data file

The double-contrast method of staining ultrathin sections with uranyl acetate and lead citrate that we used for electron tomography preferentially labels phosphate groups in nucleic acids^16,17^; therefore, the electron-dense particles in the nucleoplasm and the large particles observed in NPCs likely correspond to RNP particles, such as mRNPs or pre-ribosomes. The particles detected in NPCs were of globular shape with average dimensions of 19.7 ± 1.6 nm × 18.4 ± 1.0 nm × 19.5 ± 1.8 nm (*n* = 51), as determined by 3D bounding-boxes. Their size and globular shape, together with the strong labeling by uranyl acetate and lead citrate, suggested that these particles were pre-ribosomes. Isolated mRNPs, by contrast, form rod-shape particles of 5–7 nm diameter and variable length^12^. To test the hypothesis that these particles were pre-ribosomes, we compared them with bona fide pre-ribosomal particles trapped in the nucleus of mutant yeast strains that have a conditional nuclear export defect. The strain MNY8 carries a leptomycin B (LMB)-sensitive allele (*T539C*) of the gene encoding exportin Crm1^18^. We and others have previously reported that pre-40S and pre-60S particles are retained in the nucleus of MNY8 cells upon incubation with LMB^19–21^. Whereas the nucleoplasm of wild-type NOY505 cells (Fig. 2a) contained 75 ± 4 particles/μm^2^ (*n* = 14 cells), this number increased to 181 ± 11 particles/μm^2^ (*n* = 9 cells) in the nucleoplasm of MNY8 cells (Fig. 2b) after incubation with LMB (see quantification in Fig. 2f). The diameter and shape of these putative pre-ribosomal particles were very similar to those of the large particles observed in the NPCs (Table 2). To inhibit more specifically pre-ribosomal nuclear export, we used a temperature-sensitive mutant of the *NMD3* gene (*nmd3-2*), which encodes the adapter for Crm1 in 60S subunit precursors^22^. The *nmd3-2* mutant accumulated almost twice as many electron-dense particles in the nucleoplasm at restrictive temperature (Fig. 2c; 143 ± 8 particles/μm^2^, *n* = 9 cells) than at permissive temperature (74 ± 8 particles/μm^2^, *n* = 11 cells). We performed subtomogram averaging on these electron-dense particles, which are expected to be predominantly pre-60S particles. A consensus 3D structure of these particles was generated at 75 Å resolution (Supplementary Figure 1a). This average density map showed a flattened spherical shape similar to the electron density map of a yeast late pre-60S subunit generated at a similar resolution (Supplementary Figure 1a). In addition, both density maps accommodated the atomic structure of the yeast late pre-60S subunit along the same orientation by rigid*-*body docking. The atomic structure of a pre-40S particle did not fill-up the density map as completely as the pre-60S structure (Supplementary Figure 1b), although it should be noted that at this resolution, the large and the small subunits have similar sizes in many orientations and cannot be unambiguously distinguished^23^. These data support the assumption that most nucleoplasmic particles observed in the nucleus of the *nmd3-2* strain at the restrictive temperature are bona fide pre-60S particles. We next measured these particles and found that their size and circularity were very comparable to those of the large particles observed in NPCs in wild-type cells (Table 2). Importantly, we could readily detect such globular electron-dense particles in the nucleus of a strain bearing a temperature-sensitive allele of *RPB1* (*rpb1-1*), the gene encoding the largest subunit of RNA polymerase II (Fig. 2d). Synthesis of mRNAs is severely reduced at restrictive temperature in this mutant^24^, which we confirmed by fluorescence in situ hybridization with a poly-dT probe (data not shown). The particles detected in this strain at 37 °C were very comparable in size and circularity to the particles detected in the NOY505, MNY8, and *nmd3-2* strains (Table 2). We counted a density of 68 ± 17 particles/μm^2^ (*n* *=* 9 cells), which is close to the density determined for wild-type cells or the *rrn3-8* and *nmd3-2* mutants at 30 °C (Fig. 2f and Supplementary Table 1). These results indicate that these RNP particles do not primarily rely on RNA polymerase II for their synthesis, consistent with them being pre-ribosomes.

**Table 2:** Morphological comparison of the RNP particles found in NPCs with ribosomes and pre-ribosomal particles

Strain	NOY505 + OGP103	NOY505	NOY505	*crm1* ^*T539C*^	*nmd3-2* ^*ts*^	*rpb1-1* ^*ts*^
Particles	Cargoes in NPCs	Ribosomes	Nuclear particles	Nuclear particles	Nuclear particles	Nuclear particles
Feret diameter (nm)	18.9	19.9	19.0	18.9	19.3	19.1
SEM	0.2	0.1	0.2	0.1	0.1	0.6
Circularity	0.81	0.72	0.72	0.85	0.74	0.80
SEM	0.01	0.004	0.007	0.006	0.005	0.01
Number of particles	51	373	108	215	315	265
Number of cells	37	10	10	8	9	9

To confirm that the particles in NPCs were pre-ribosomes, we investigated whether the presence of these particles required ribosome biogenesis by observing a thermosensitive mutant of gene *RRN3*, which encodes an RNA polymerase I-specific transcription factor^25^. A pronounced defect in rRNA synthesis at restrictive temperatures was previously observed in the temperature-sensitive *rrn3-8* mutant^26,27^. As expected if RNA pol I transcription is defective^28^, the nucleolus was fragmented and detached from the nuclear envelope in this mutant at the restrictive temperature (Fig. 2e, black dotted lines). The ribosome density in the cytoplasm in the *rrn3-8* mutant, measured in a zone of 250–300 nm around the nuclear envelope, fell from 851 ± 152 ribosomes/μm^2^ (*n* = 20 cells) at permissive temperature to 487 ± 115 ribosomes/μm^2^ (*n* = 17 cells) at restrictive temperature, attesting to the strong defect in ribosome production (Supplementary Table 1). Similarly, the density of electron-dense globular particles in the nucleoplasm in the *rrn3-8* mutant at 37 °C (47 ± 13 particles/μm^2^, *n* = 8 cells) was significantly lower than at 30 °C (73 ± 13 particles/µm^2^, *n* = 10 cells) (Supplementary Table 1). Under these conditions of strongly reduced ribosome biogenesis, the number of particles translocating through the NPC fell from 6.2 particles/100 NPCs in NOY505 wild-type cells (35 particles in 566 NPCs counted in 105 cells) to 1.8 particles/100 NPCs (4 particles in 218 NPCs counted in 57 cells) in the *rrn3-8* strain at non-permissive temperature. We saw no example of two particles per NPC in *rrn3-8* cells at the restrictive temperature. We conclude from this and the other structural and functional data described above that a large majority of the particles detected by electron tomography in the NPCs were pre-ribosomal particles.

### Pre-ribosome distribution reveals the path through the NPC

**Fig. 3:**
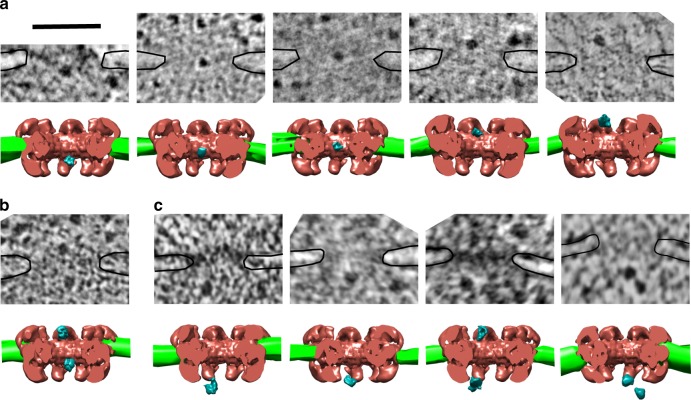
3D-rendering of pre-ribosomal particles within the NPC. **a** The yeast NPC model was docked into subtomograms showing pre-ribosomal particles in various regions of the NPC in NOY505 or OGP103 cells. The gallery displays tomographic sections and the corresponding 3D models. Pre-ribosomal particles are segmented in blue, the nuclear envelope in green and the docked NPC model is shown in red. **b** An example of NPC containing two pre-ribosomal particles. **c** In *nmd3-2*^*ts*^ cells at 37 °C, most pre-ribosomal particles were found at the nuclear ring or below. Black lines indicate the nuclear envelope. Scale bar: 100 nm

Due to lack of staining, the mass of the NPCs was not visible in the tomograms. To visualize the position of pre-ribosomes in NPCs, we superimposed our data with a model of the yeast NPC recently established by cryo-EM^7^. We checked that the largest distance between the bended edges of the nuclear envelope in these subtomograms was equal to 100 ± 5 nm, which is comparable to similar measurement performed in yeast^6,29,30^. In addition, the width of the nuclear envelope in these subtomograms was estimated to 31 ± 3 nm, which matches the width of the nuclear envelope previously established by cryoelectron microscopy^6^. The combination of these two measures confirmed that the section plane in these subtomograms was orthogonal to the nuclear envelope and contained the central axis of the NPC. Based on this conclusion, coordinates were attributed to each particle found in NPCs by measuring its distance from the central axis (*X*) and from the median plane (*Y*) of the NPC, the latter being defined by the bended edges of the nuclear envelope (Supplementary Figure 2). For 3D rendering, the structure of the yeast NPC^7^ was positioned in the subtomograms such that the cytoplasmic ring was attached to the outer nuclear membrane and the nuclear ring to the inner nuclear membrane (Fig. 3). Figure 3a shows a representative gallery of pre-ribosomal particles located at different positions on the path to the cytoplasm.

**Fig. 4:**
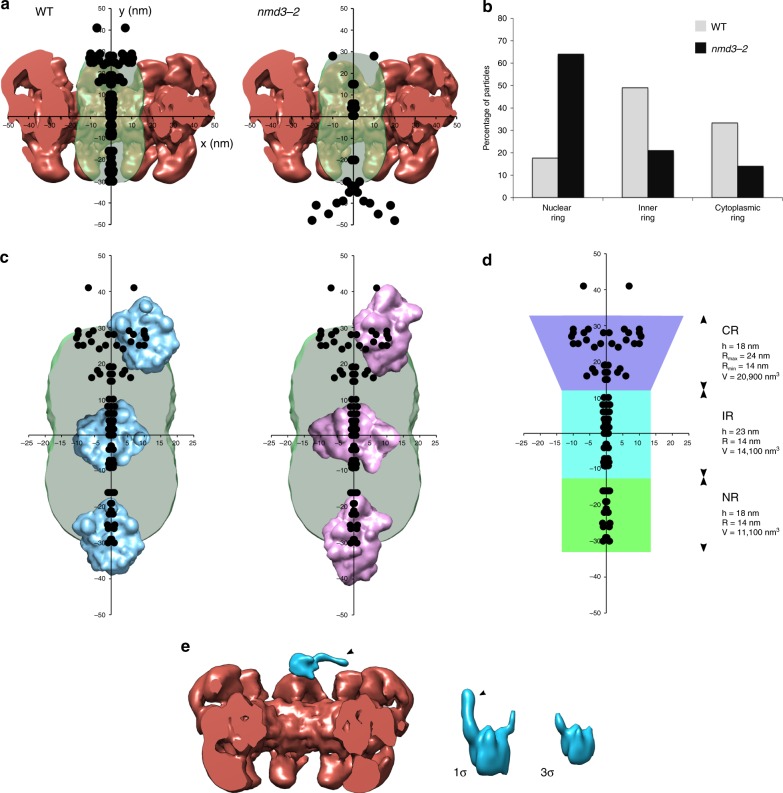
The path of the pre-ribosomes across the NPC. **a** Positions of pre-ribosomes in NPCs in wild-type cells (WT) and pre-60S particle nuclear export-defective mutant *nmd3-2*. The dots represent the centroid of the particles relative to the median plane section (*X*) and the central axis (*Y*) of the NPC. The distribution was mirrored along the *Y*-axis for the representation. **b** Distribution of pre-ribosomal particles in the nuclear, inner, and cytoplasmic rings in wild-type and *nmd3-2* cells. **c** The central transporter was superimposed with the distribution of pre-ribosomal particles as in **a** and with pre-60S (blue) or a pre-40S (pink) particles at the same scale. The central transporter is large enough to accommodate pre-ribosomes whatever their orientation. The path of the particles is constrained in a 25–30 nm diameter channel at the center of the nuclear and inner rings. **d** The volume of the space explored by pre-ribosomal particles was estimated in the nuclear, inner, and cytoplasmic rings the NPC. For the diameter of the central channel, we took into consideration the longest dimension of a pre-ribosomal particle, i.e. 28 nm for a pre-40S particle^65^. **e** A representative 3D reconstruction of a pre-ribosome at the cytoplasmic ring showing additional densities pointing towards the NPC, almost perpendicular to the central axis (black arrow). On this example, segmentation was performed by isosurfacing with a gray-level cut-off of 1*σ* but the additional density is still detected with 3*σ* cut-off (*σ*: standard deviation of the gray-level distribution). Source data for **a** and **b** are provided as a Source Data file

As shown in Fig. 4a–c, the 51 particles located in NPCs of NOY505 or OGP103 cells were detected in the nuclear ring (*Y* = −21.5 ± 5.8 nm; S.D.), the inner ring (*Y* = 1.2 ± 6.1 nm), and the cytoplasmic ring (*Y* = 25.1 ± 6.8 nm). Strickingly, all the particles were located within the central transporter of the NPC (Fig. 4a, c). In the nuclear and inner rings, the pre-ribosomes were very close to the NPC central axis: the centroid of the pre-ribosomes was distant of 0.6 ± 0.3 nm (SD) and 0.7 ± 0.6 nm from NPC central axis, respectively. Considering that the mean diameter of the pre-60S subunit is ~25 nm, we estimate that the particles explore a cylindrical space of ~26–28 nm in diameter. In the cytoplasmic ring, by contrast, the pre-ribosomal particles are shifted 5.4 ± 3.7 nm from the central axis of the NPC. Thus, the distribution of the particles defines two compartments within the central transporter: a narrow channel in the nuclear and inner rings and a wider conical space on the cytoplasmic side (Fig. 4d). Their off-axis position suggests that the particles emerging from the central channel, likely associated with nuclear transport factors, interact with the asymmetric Nups located on the cytoplasmic side of the NPC. Interestingly, we noticed one or two additional elongated densities attached to some pre-ribosomal particles on the cytoplasmic side of the NPC (Fig. 4e). In the case of NPCs containing two particles, we observed these additional densities only on the particles located on the cytoplasmic side. They were not observed either on particles in the nucleoplasm or on mature ribosomes in the cytoplasm, regardless of the gray level threshold (Supplementary Figure 3), indicating that they were not artifacts of the tomographic reconstruction. Because of their particular orientation and localization, we speculate that these densities might be due to extended structures formed by cytoplasmic Nups that interact with the transported pre-ribosomal particles.

### The central transporter constitutes a permeability barrier

In NOY505 or OGP103 cells, the proportion of particles in NPCs was higher in the inner ring (49%), than in the nuclear ring (18%) or the cytoplasmic ring (33%) (Fig. 4b). This higher proportion of particles in the inner ring was even more pronounced when restricting the analysis to NPCs containing a single particle: 15% were found on the nuclear side, 64% in the inner ring, and 21% on the cytoplasmic side (Mann–Whitney *U*-test, *p* < 0.0006 for nuclear vs. inner ring, and inner vs. cytoplasmic ring). The higher proportion of particles in the inner ring could not be merely explained by a longer path or a larger volume of the space occupied by the particles in this zone when compared to the nuclear and cytoplasmic rings (Fig. 4d). When two particles were present in the same NPC (7 NPCs), they were found on either side of the inner ring (Fig. 3b), except in one case where one of the two particles was present in the inner ring. This suggests that the core of the central channel cannot accommodate two particles simultaneously. This distribution of the pre-ribosomal particles supports the hypothesis that the particles translocating through the NPC spend more time in the inner ring than in the nuclear or cytoplasmic rings. The distribution was very different in the *nmd3-2* mutant at non-permissive temperature, when Crm1 recruitment to the pre-60S particles is defective (Figs. 3c and  4a, b). As an exportin of pre-60S particles, Crm1 is expected to interact with FG Nups and thereby lower the energy necessary for crossing the barrier constituted by FG Nups in the NPC. Strickingly, the largest fraction (64%) of the particles associated with NPCs in the *nmd3-2* mutant at 37 °C was found in the nuclear zone, 30–50 nm away from the nuclear envelope, outside of the central transporter in the space occupied by the nuclear basket (Fig. 4b). Most of these particles did not line up on the central axis of the NPC. Several NPCs displayed two pre-ribosomes in the nuclear zone (see example in Fig. 3c), a situation not observed in wild-type cells. Some particles were detected within the central transporter or at the cytoplasmic side and may correspond to pre-40S particles. These data indicate that Crm1 is required for entry into the central transporter, but not introduction into the nuclear basket. We conclude that the permeability barrier overcome by Crm1 starts at the nuclear side of the central transporter.

### Estimation of the dwell time of pre-ribosomes in the NPC

**Fig. 5:**
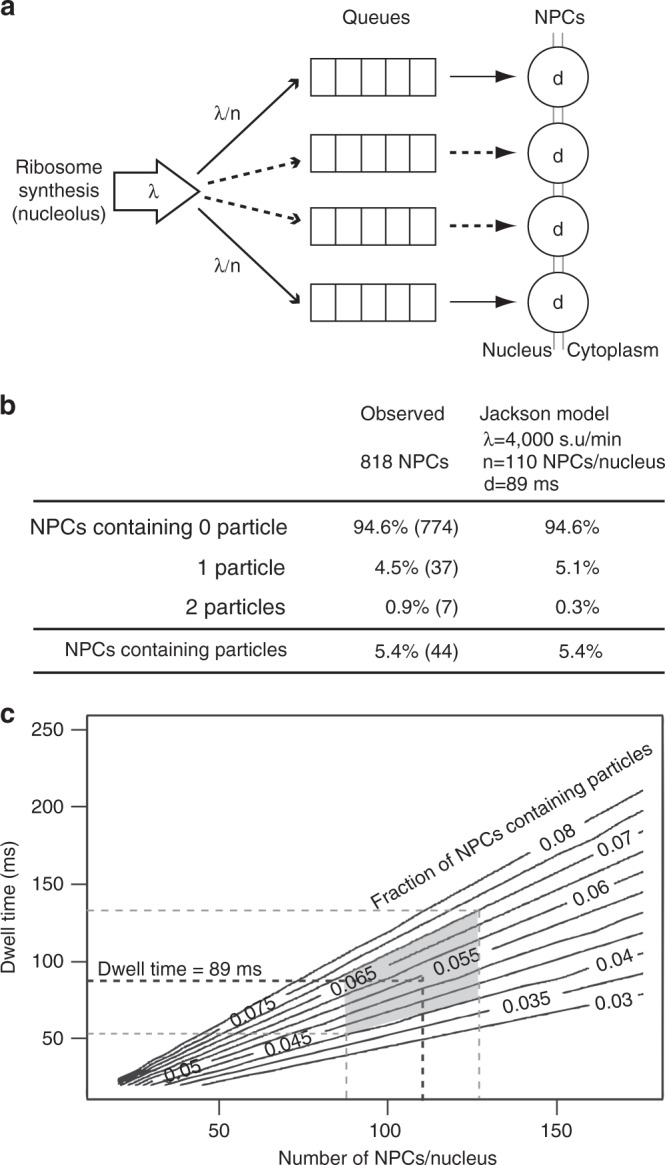
Determination of the dwell time of pre-ribosome in NPCs by a probabilistic method. **a** Nuclear export of pre-ribosomes was modeled according to a Jackson queueing network. Each NPC is characterized by a queue and a dwell time. We assume that pre-ribosomes are routed to NPCs with equal probability, which is supported by the even distribution of pre-ribosomes among nucleolar and nucleoplasmic NPCs. **b** Based on an average of 110 NPCs per cell and a flux of 4000 pre-ribosomal particles/min, the best fit of the model to the experimental data (5.4% of occupied NPCs) is obtained for a dwell time of 89 ms. The values predicted by the Jackson model correspond to NPCs with no particle, containing one particle, or occupied by one particle with one particle in the queue. **c** The robustness of this estimated dwell time was tested considering combined errors in the average number of NPCs (110 ± 20) and the observed NPC occupancy rate (5.4 ± 1.5%). The gray polygon delimits the domain defined by these values. The dwell time in this domain ranges from 55 to 140 ms

Ultra-fast freezing of the cells takes place in <10 ms^15^. This time scale is less than the translocation time of large RNP particles, as shown by live cell analyses of mRNP nuclear export in yeast^31^. Therefore, the proportion of NPCs containing a pre-ribosomal particle in the tomograms should reflect the actual proportion (on average) of occupied NPCs at any given time in exponentially growing yeast. Following this assumption, we performed a probabilistic analysis of the tomograms in order to infer the dwell time of pre-ribosomes in NPCs. We modeled nuclear export of pre-ribosome particles according to a Jackson network queuing model^32^. This probabilistic model has been widely used to describe and optimize networks in various fields like telecommunication, transportation, or health care. Here, NPCs were approximated to a network of parallel nodes, each characterized by a processing time (dwell time) and a queue of exported cargoes (Fig. 5a). At equilibrium, the probability that a NPC is occupied by a cargo depends on the flux of cargoes, the total number of NPCs and the dwell time. Ribosome synthesis in *S. cerevisiae* was previously estimated at 2000 ribosomes/min during the exponential growth phase^33^, which implies export of 4000 subunits/min from the nucleus. We made the approximation that the flux of pre-ribosomes is constant throughout the cell cycle. The number of NPCs per nucleus was found previously to vary from ~80 in G1 phase to ~120 in S phase and ~140 in mitosis^34^. Given that yeast cells growing exponentially are mostly in G1 and S phases^35^, we considered an average of 110 NPCs per cell. Connecting equilibrium occupation to dwell time also requires to assume that the flow of pre-ribosomal particles is unidirectional from the nucleus to the cytoplasm, and that all pre-ribosomal particles (pre-40S and pre-60S) behave identically when crossing the NPC. Applying these assumptions, the best fit to a Jackson network queuing model for an NPC occupancy rate of 5.4%, as measured in the tomograms, was obtained with a dwell time of 89 ms (Fig. 5b). Under these conditions, the queuing model also predicted that only a small fraction of the NPCs (0.3%) should have a queue and that these queues should not contain more than one particle. Thus, according to this model, pre-ribosomes are unlikely to compete with one another for entry into an NPC. The NPCs in which we observed two particles simultaneously in the channel of the pore might reflect the residual queuing predicted by the model. To test the robustness of this predicted dwell time, we considered the effects of possible inaccuracies in the measurement of NPC occupancy made from the tomograms (Fig. 5c). When the NPC occupancy rate was varied from 0.04 to 0.07 (i.e. ~25% variation around the measured value of 0.054), the dwell time varied from 65 to 115 ms. Similarly, varying the average number of NPCs from 90 to 130 per cell changed the dwell time to 75–110 ms. Taking the error in these two parameters together, the estimated dwell time remained between 55 and 140 ms. Thus, we conclude with reasonable confidence that translocation of pre-ribosomal particles through the NPC takes, on average, ~90 ± 50 ms.

## Discussion

**Fig. 6:**
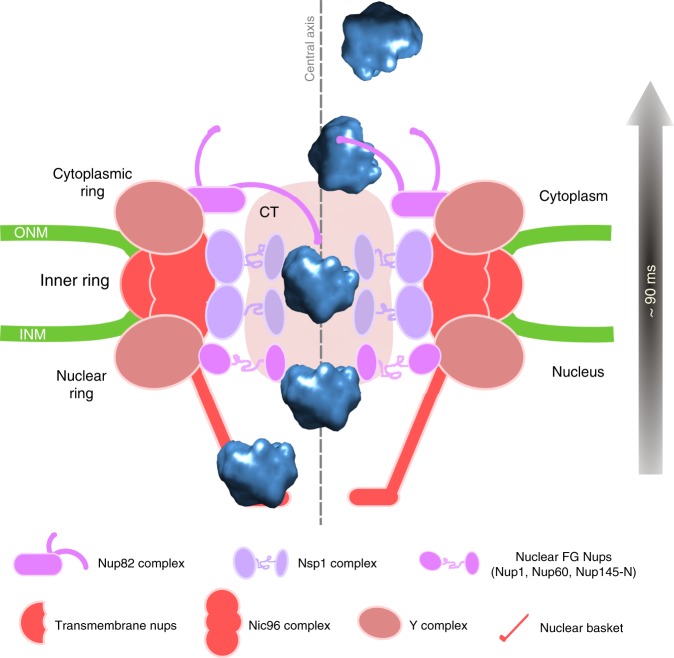
Model of the translocation of pre-ribosomal particles through the NPC. A pre-60S ribosomal particle crossing the NPC is represented. After transient interaction with the nuclear basket, pre-ribosomes are rapidly transferred to the central transporter, which constitutes the main permeability barrier. The path of the particles is constrained in a narrow channel along the central axis as they cross the nuclear and inner rings. Pre-ribosomes spend a longer time in the inner ring than in the nuclear or cytoplasmic rings, maybe due to the high density of FG Nups in the inner ring (especially the Nsp1/Nup49/Nup57 complex), which provides multiple contact sites for pre-ribosomes. We postulate that extraction from this narrow channel at the exit of the inner ring involves interaction with asymmetric Nups found at the cytoplasmic ring, like the Nup82 complex. Disassembly of the nuclear transport factors then makes transport irreversible. This path is consistent with the requirement of the Nsp1 and the Nup82 complexes for pre-ribosome translocation. The NPC and the pre-ribosome are drawn at the same scale. CT central transporter. Modeling of the pre-60S particle is based on PDB structure 5H4P [https://www.rcsb.org/structure/5H4P])

In this study, we could detect single native RNP particles of globular shape in transit in the NPC using electron tomography. The analysis of the distribution of these particles relative to the structure of the NPC informs us on the topology of their path through the NPC and leads us to define several steps in the route to the cytoplasm (Fig. 6). We identified these particles as pre-ribosomes based on their morphological resemblance with the particles that accumulate in the nucleoplasm of the *crm1* and *nmd3* mutant strains, which are defective in pre-ribosome nuclear export, and also because the number of these particles in the NPCs fell by 70–80% in a *rrn3* mutant strain, in which RNA polymerase I transcription is deficient. In addition, a large fraction of these particles accumulates at the nuclear entry of the NPC upon loss of function of Nmd3, the adapter of exportin Crm1 on pre-60S particles. We cannot exclude that some of the particles are mRNPs; however, the average apparent diameter (~20 nm), the homogeneous size, and the globular shape of the particles do not correspond to the rod-like structure observed for purified mRNPs in yeast^12^, and recently proposed for human mRNPs studied by proximity ligation detection assays^36^. In addition, single mRNP tracking experiments in human cells have concluded that mRNPs unwind during their passage through the NPC^37^. In contrast, the highly ordered structure of pre-ribosomes before their nuclear export, as revealed by single particle cryo-electron microscopy^38,39^, indicates that pre-ribosomes cross the NPC in a compact conformation, consistent with our observations.

Our data indicate that pre-ribosomes exit the nucleus equally often through all NPCs, irrespective of their proximity to the nucleolus, suggesting that none of the NPCs are specialized for pre-ribosome export. The absence of proteins Mlp1 and Mpl2 in the nuclear basket of nucleolar NPCs^40,41^, or the occurrence of the late nuclear maturation steps of pre-ribosomes in the nucleoplasm have led to speculate that some NPCs might specifically intervene in pre-ribosome nuclear transport. Our observations rather prompt us to propose that pre-ribosomal particles diffuse around the nucleoplasm and the nucleolus before nuclear export, as also proposed for mRNPs^41^. This conclusion is supported by the theory of diffusive transport through narrow channels^42^: due to the small area occupied by NPCs in the nuclear envelope and the large volume of the nucleus, a particle is likely to explore a large fraction of the nucleus by diffusion before it reaches an NPC and exits the nucleus, irrespective of its synthesis site. Randomness of the exit NPC could be further favored by competition with other cargoes for NPC binding and entrance, which may force a pre-ribosome to try several NPCs before finding the exit way. While the queueing model used here predicts that pre-ribosomal particles are unlikely to compete with one another at this step, it does not rule out competition with the multiple other cargoes exported from the nucleus.

Passage through the NPC has been proposed to occur through both a central conduit and peripheral channels^9,29^. The structure of the central conduit of the NPC remains poorly defined, and is observed in cryo-EM reconstruction as a dense structure called the central transporter^6,9^. We find that all the pre-ribosomal particles detected in the NPCs of wild-type strains are located in the central transporter. The distribution of the particles reveals that they follow a 25–30 nm wide channel in the center of the central transporter, at the level of the nuclear and inner rings. The exit of this narrow channel occurs at the junction between the inner and the cytoplasmic ring, where the space occupied by the particles rapidly enlarges. Importantly, while the scaffolds of the cytoplasmic and nuclear rings display a high degree of symmetry, the asymmetry of the space occupied by the particles indicates that the FG-Nups found on the two sides of the NPC adopt different organizations. Consistent with the central route of the preribosomes observed here, the passage of both pre-40S and pre-60S particles is known to be strongly affected by mutations in Nsp1p, a major component of the central channel, whereas it is much less sensitive to mutations in the Nups that form the Y complex, which organizes the periphery of the inner ring^43,44^. In contrast to pre-ribosomal particles, mRNP export in yeast is deficient in mutants of the Y complex and is not affected by most mutations in Nsp1p^43,45^. This suggests that pre-ribosomes and mRNPs follow different paths through the NPC. Consistent with this, tracking fluorescent single mRNPs led to the conclusion that they interact primarily with the periphery of the NPC^8^. Immuno-EM studies of Dbp5 and Gle1, however, rather indicated that most mRNPs follow a central path through the NPC^29^. Thus, the question of the route taken by mRNPs through the NPC remains open. The giant Balbiani ring mRNPs in Chironomus cells do follow the central axis of the NPC during their export from the nucleus^10,11^. Similarly, studies of the nuclear import of large cargoes in metazoan cells by electron microscopy, including viruses^46^ and protein-coated gold particles^47,48^, also showed that these particles pass through the central axis of the NPC. This axial translocation pathway of large cargoes may be primarily explained by topological constraints imposed by their bulky structures, which may be also the case for pre-ribosomes.

Our data suggest distinct steps during nuclear export of pre-ribosomes. The expected interaction with the nuclear basket appears to be very transient under wild-type conditions, since we only noticed pre-ribosomes accumulating in the nuclear basket area in the *nmd3-2* mutant. This step may not require Crm1, but could involve other pre-ribosomal nuclear export factors like Mex67/Mtr2. Second, the transfer of the cargoes to the central transporter requires the passing of a stringent energy barrier, which is then primarily mediated by Crm1. Once introduced into the central channel, the particles find the most energetically favorable zone in the inner ring, as indicated by the longer dwell time of the cargoes in this zone. This might be explained by a high density of contacts with FG Nups, especially the Nsp1 complex. Exiting the central channel could then involve high-affinity interactions with the asymmetric Nups located on the cytoplasmic ring, like Nup159, which would displace the particles out of the central channel. Transfer to the cytoplasmic ring would then be made irreversible by dissociation of the transport factors. Consistent with this model, nuclear export of pre-ribosomes requires the Nup82 complex^43^, which is anchored to the cytoplasmic ring and includes Nup82, Nup159, Dyn2, and Nsp1^49^. This complex provides binding sites for transport factors and forms a platform for the final steps of mRNP export^50,51^. The human homolog of Nup159, Nup214, contains high-affinity binding sites for exportin Crm1^52,53^ and is required for Crm1-mediated nuclear export^54^. It was recently proposed that the Nup82 complex projects towards the central axis of the NPC rather than towards the cytosol^7,49,50^. The off-axis position of the particles observed in our study is consistent with this model, which places the Nup82 complex in ideal position to bind pre-ribosomes and favor their exit from the central channel. The additional densities that we observed on pre-ribosomes in the cytoplasmic ring (Fig. 4e and Supplementary Figure 3) may correspond to such interactions. Intriguingly, the GLFG repeats containing domain of Nup116, a nucleoporin of the cytoplasmic ring, were also shown to extend into the center of the NPC, suggesting that they move in and out of the inner ring^55^; they could thus interact with cargoes as they traverse the central channel and orient their transfer towards the cytoplasmic side of the NPC.

By using a Jackson queuing network to model nuclear export, we infer that the mean dwell time of pre-ribosomes within the NPC is in the range of 90 ms. This value compares well with those measured recently for mRNPs in *C. tentans* and in budding yeast by means of fluorescence microscopy of live cells^31,56^, although shorter dwell times have also been reported in vertebrates^8^. Beyond technical issues regarding data analysis and the drawbacks of mRNP tagging discussed previously^13,14^, differences in the dwell time may arise from the size and state of compaction of the cargoes (different mRNPs were used in these experiments), as well as the density of export receptors on them, as has been shown with functionalized quantum dots^57^. Quality control of mRNPs at the basket of the NPC may also increase the dwell time on the nuclear side, whereas no quality control mechanism has been described for pre-ribosomes at the NPC. Moreover, the scale and resolution of electron tomography are not the same as those of single-particle tracking by fluorescence microscopy. Here, our study only takes into account particles detected in the central transporter and therefore focuses on the passage of RNP particles through the core scaffold. In contrast, much larger cytoplasmic and nuclear zones, up to 150 nm away from the fluorescently labeled transmembrane nucleoporin POM, were considered in the studies of mRNPs by fluorescence microscopy. Observation of pre-ribosomes and mRNPs with similar techniques is required to compare more directly the dynamics of these two classes of RNP particles. Finally, it should be noted that our estimate of the dwell time of pre-ribosomal particles in NPC did not consider any potential competition for binding to the NPC by non-ribosomal RNAs and proteins. In that respect, a 90 ms dwell time may correspond to a high estimate, as a lower number of NPCs available to pre-ribosomal particles would lead to the calculation of a shorter dwell time (Fig. 5c). Whether and how such a competition affects pre-ribosomal transport remains an open question.

We conclude from this work that pre-ribosomal particles are funneled through the NPC in a compact conformation and follow the central axis in the nuclear and inner rings. The electron tomography approach used here allows the observation of native RNP particles and can be combined with the mutational analysis of the nuclear transport machinery to refine the structural and biophysical models describing the translocation of cargoes through the NPC. Increasing the throughput and the resolution of this method could potentially give access to the orientation of pre-ribosomes within the NPC or to differences in the nuclear export of the pre-40S and pre-60S particles.

## Methods

### Strains

The following strains of the yeast *S. cerevisiae* were used in this study: NOY505^58^, MNY8^18^, *nmd3-2*^*ts*^^22^, *rrn3-8*^*ts*^^26^, *rpb1-1*^*ts*^^24^ (kindly provided by Dr. Françoise Stutz, University of Geneva) and OGP103 (unpublished; generous gift from Dr. Olivier Gadal, LBME, Toulouse). NOY505 is a wild-type strain previously used for studying the nucleolus. The OGP103 strain was constructed from strain TAK201^59^, a strain derived from NOY397^58,60^ like NOY505. In strain OGP103, the ribosomal genes are interspersed with *LacO* repeats to tag the rDNA locus on chromosome XII, which does not modify the nucleolar morphology or ribosomal RNA processing and is irrelevant for this study. OGP103 and NOY505 cells display similar nucleolar morphology and growth rate.

### Sample preparation

All strains were grown to mid-log phase in complete YP media with 2% glucose. To trigger pre-ribosomal subunit accumulation in the nucleus, MNY8 cells were treated with 100 nM leptomycin B (LC Laboratories) for 45 min and *nmd3-2*^*ts*^ cells grown at 30 °C were shifted to 37 °C for 2 h. To interrupt RNA polymerase II activity, the *rpb1-1* thermosensitive strain was shifted from 25 to 37 °C for 60 min. For specific inhibition of RNA polymerase I activity, the *rrn3-8* thermosensitive strain was shifted from 25 to 37 °C for 90 min. For ultrafast freezing, yeast cells were harvested by filtration on nitrocellulose membranes with 0.45 μm pores (Millipore) or by centrifugation at 3000 rpm for 3 min using 1.5-ml microcentrifuge tube (Eppendorf). Yeast paste was mixed with liquefied low-melting-point agarose (Sigma) just before high-pressure freezing in a Leica EMPACT. Frozen cells were then transferred to an automated freeze-substitution unit (AFS2, Leica) for freeze substitution in anhydrous acetone containing 0.01% osmium tetroxide, 0.1% glutaraldehyde, and 0.1% uranyl acetate at −90 °C. The temperature was gradually raised to −50 °C over the course of 3 days. The samples were embedded in Lowicryl HM20 and the resin was cured under UV light at −35 °C. Sections of a nominal thickness of 80 nm were cut on a UCT ultramicrotome (Leica) and picked up on 150-mesh formvar-carbon-coated copper grids or collodion carbon-coated rhodium-plated copper grids (Electron Microscopy Science). Ten nanometer protein A-gold conjugates (Aurion) were added to the surface of each section to serve as fiducial markers for subsequent image alignment in IMOD^61^. Sections were stained with lead citrate and 5% uranyl acetate in 70% methanol.

### Electron tomography

Single-axis tilt series were recorded on a JEM 2100 or a JEM 1400 transmission electron microscope (JEOL) operated at 200 or 120 kV, respectively. The tomography plug-in of the Digital Micrograph software (Gatan) was used to acquire images automatically every 2° over a ±60° range using an Ultrascan 2K × 2K CCD camera (Gatan) at a pixel size of 1.01 nm (JEM 2100) or a Gatan Orius SC1000B camera at a pixel size of 0.64 nm (JEM 1400) at a magnification of ×10,000. Tomograms were reconstructed with IMOD by the simultaneous iterative reconstruction technique (SIRT). The nominal resolution in our tomograms is estimated at about 4 nm according to the Crowther criterion^62^.

### Image analysis

To assess the morphology of the particles in tomograms, thresholding segmentation was performed in two dimensions on several tomographic sections and standard measurement option in ImageJ software was used to determine shape parameters, including circularity and Feret’s diameter. Size and circularity filters of 150 nm^2^ and 0.6, respectively, were applied to the segmented particles to exclude particles much smaller than pre-ribosomes or of non-globular shape. Measurements of particles in the nucleus or the nucleoplasm were performed on 8–10 cells for a total of 108–373 particles per yeast strain (Supplementary Table 1). Nuclear and cytoplasmic particle counting was performed using the ImageJ cell counter plugin in 8–14-cell sections. Measurements were performed on 96 to 1629 particles over a cumulative surface ranging from 1.86 to 4.6 μm^2^ (Supplementary Table 1).

To measure the position of pre-ribosomal particles within NPCs, we defined the centroid of a particle as the center of the circle framing this particle. Coordinates were attributed to each particle in a spatial system defined by the line joining the bended edges of the nuclear envelope (median plane of the inner ring) and by the central axis of the NPC (Supplementary Figure 2). The coordinates were established by measuring the distances between these axes and the centroid of each particle using the free hand line tool in ImageJ.

### Morphological comparison of the RNP particles found in NPCs

For Table 2: Ferret’s diameter and circularity were evaluated on the central section of the particles observed in tomograms. In wild-type cells, measurements were performed on the large globular particles seen in the NPCs, on ribosomes in the cytoplasm, and on nucleolar particles, which we assume to be pre-ribosomes. In *crm1*^*T539*^ cells (MNY8 strain), measurements were performed on the particles accumulated in the nucleus after treatment with 100 nM LMB for 45 min, which prevents nuclear export pre-ribosomes. In *nmd3-2*^*ts*^ cells, measurements were performed on the particles blocked in the nucleus after a 2 h shift to 37 °C, which are expected to be pre-60S particles. In *rpb1-1*^*ts*^ cells, measurements were performed on the particles detected in the nucleus after cessation of mRNA synthesis in cells shifted for 1 h to 37 °C. SEM: standard error of the mean.

### Subtomogram averaging

Averaging of putative pre-60S particles in subtomograms was carried out with the Automated Recognition of Geometries, Objects, and Segmentations (ARGOS) software package (FEI Company). A volume of ~0.045 μm^3^ (1684 × 589 × 45 voxels) was extracted from a tomogram of the nucleus of an *nmd3-2* cell at 37 °C using the IMOD *trimvol* command. The trimmed volume contained several pre-ribosomal particles in the nucleus. A geometric template of 24^3^ voxels was first used by the ARGOS software to conduct a template matching and generate an average 3D structure. The average structure was then used as a reference and the entire procedure was repeated several times to refine the average structure. The low-resolution density map of the yeast 60S subunit^63^ (pdb accession code: 4V7R [http://www.rcsb.org/structure/4V7R]) was generated with the *molmap* command in UCSF Chimera^64^. Fitting of the 60S atomic structure into these density maps, or of one density map into another, was performed using the global search option of the USCF Chimera command *FitMap*.

### Docking of yeast NPC

When a large globular particle was unequivocally observed within an NPC, a subtomogram centered on this NPC was extracted by using the IMOD *trimvol* command. The nuclear envelope was hand-segmented using drawing tools in IMOD. The volume corresponding to the particle was displayed by isosurface thresholding in IMOD. The structure of yeast NPC (emdb accession code: 7321) was docked in the subtomogram as shown in Fig. 3. The volume of the yeast NPC was modified by using the *Volume Eraser* tool in UCSF Chimera. NPC docking on segmented nuclear envelope was performed manually in UCSF Chimera.

### Statistical analyses

The Shapiro–Wilk Normality Test was performed before using one-way analysis of variance (ANOVA) for independent samples; differences were considered significant at *p* < 0.05. Concerning the spatial distribution of pre-ribosomal particles within the NPC, the non-parametric Mann–Whitney *U*-test was used (H0 null hypothesis: two samples come from the same population). The number of experiments and replicates is mentioned in the figure legends.

### Jackson’s queueing model

To model the nuclear export of pre-ribosomes through NPCs, we used a Jackson network of *n* distinct M/M/1 queuing processes (see Fig. 5a). The pre-ribosomes are seen as customers arriving in the system (the cell) according to a Poisson process with rate *λ* (flux of cargoes estimated at 4000 subunits/min). Each NPC plays the role of a server with exponential service time and a mean dwell time *d*. No limitation is given on the queue size in front of each server. Assuming a uniform routing of customers between the servers, we know (for example, see ref. ^32^) that the occupation rate of each queue is equal to *ψ* = *λd*/*n* and that the equilibrium distribution of the number *N* of customers in a given queue is *P*(*N* = *k*) = *ψ*^*k*^(1 − *ψ*). We chose the value of d that gave us the measured NPC occupancy rate of 5.4%. Changes in the values of *n* and *d* gave the level lines plotted in Fig. 5c.

### Reporting summary

Further information on experimental design is available in the Nature Research Reporting Summary linked to this article.

## Supplementary information

Supplementary Information

Reporting Summary

Source Data

## Data Availability

The data that support the findings of this study is available from the corresponding author upon reasonable request. The source data underlying Figs. 2 and 4, Table 2, and Supplementary Table 1 are provided as a Source Data file.
