# Supplementary material for: The path of pre-ribosomes through the nuclear pore complex revealed by electron tomography
Source: Nat Commun. 2019 Jan 30;10:497. doi: 10.1038/s41467-019-08342-7 (PMC6353910; doi:10.1038/s41467-019-08342-7)
Supplement: Supplementary file 1 — Supplementary Information [file 41467_2019_8342_MOESM1_ESM.pdf]

SUPPLEMENTARY INFORMATION

The path of pre-ribosomes through the nuclear pore complex revealed by electron tomography

Delavoie et al.

		Cells	Particles	Cumulative surface (micron^2)	Density per micron^2	SEM		
nucleoplasm	**wt**	14	335	4.56	75	4	p < 0.001	p > 0.05
	**MNY8 + LMB**	9	552	2.71	181	32		
	***rpb1-1* (37°c)**	9	265	3.40	68	17		
	***nmd3-2* (30°c)**	11	224	3.06	74	3	p < 0.001	
	***nmd3-2* (37°c)**	9	402	2.80	143	8		
	***rrn3-8* (30°c)**	10	148	2.02	73	13	p < 0.0025	
	***rrn3-8* (37°c)**	8	96	2.03	47	19		
cytoplasm	***rrn3-8* (30°c)**	10	1629	1.94	851	152	p < 0.001	
	***rrn3-8* (37°c)**	8	849	1.86	487	113		

Supplementary Table 1. Quantification of electron-dense particles in the nucleoplasm and ribosomes in the cytoplasm of wild-type and mutant yeast strains. Counting was performed on *nmd3-2^{ts}*, *rrn3-8^{ts}* and *rpb1-1^{ts}* thermosensitive cells shifted to non-permissive temperature (37°C) for 2 h, 90 min and 60 min, respectively. The MNY8 strain bears the *crm1-T539C* allele sensitive to leptomycin B (LMB) and was treated for 45 min with this compound. These data are partially displayed in the graph shown in Fig. 2f. Statistical significance of the results was assessed by applying a one-way ANOVA test. The source data for this table are provided as a Source Data file.

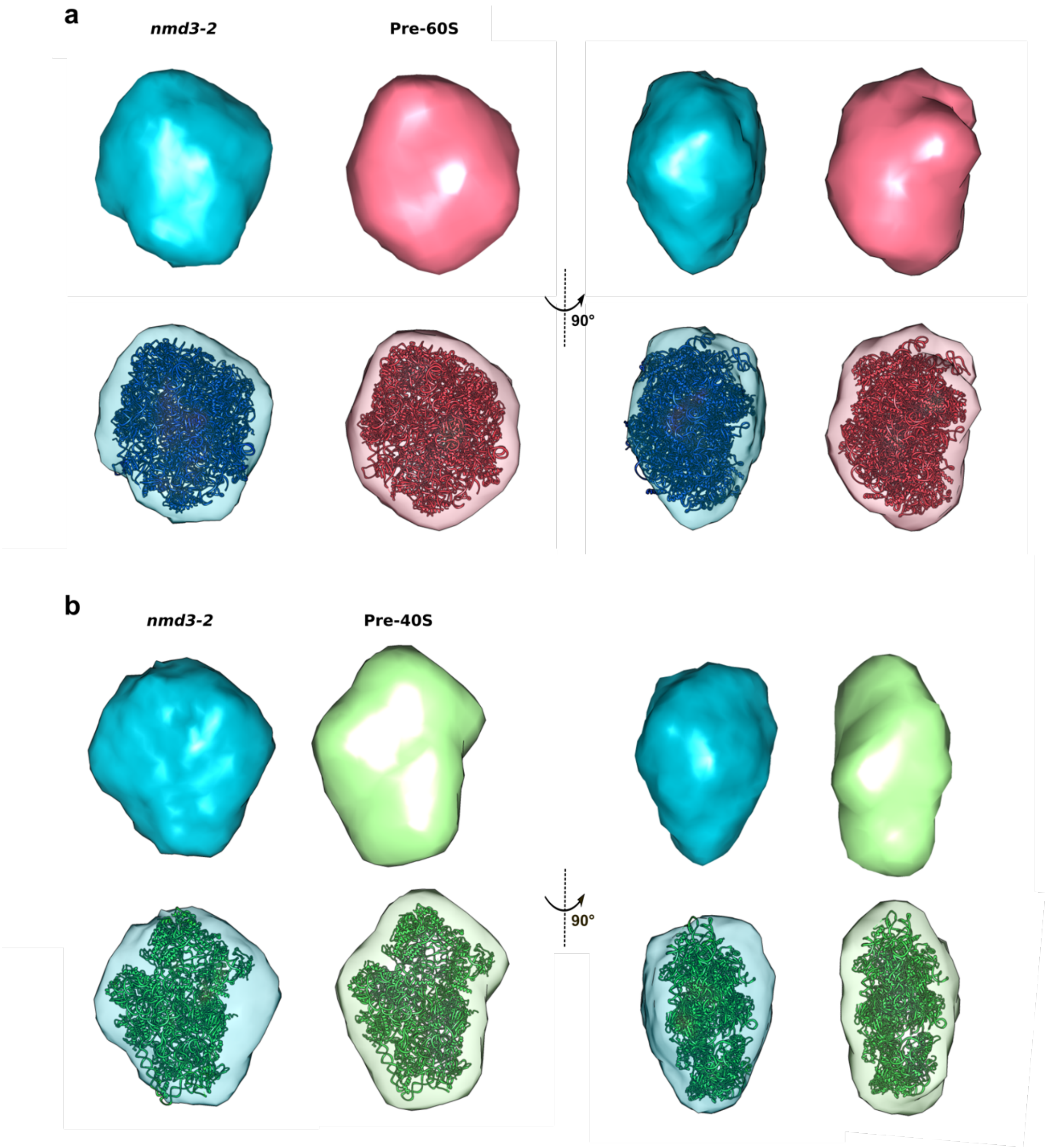

Supplementary Figure 1. Subtomogram averaging of electron-dense particles accumulating in the *nmd3-2*^{ts} strain at 37°C. A low-resolution density map obtained by subtomogram averaging of electron-dense particles found in the nucleus of *nmd3-2*^{ts} cells after 2 hours at 37°C is displayed in blue. The resolution of this reconstruction is 75 Å according to the 0.5-criterion of the Fourier shell correlation plot. For comparison, density maps of yeast pre-60S (pink) or pre-40S (green) particles were generated from PDB structures 5H4P and 6EML, respectively, at 75 Å resolution by frequency filtering. The same structures of pre-60S and pre-40S particles at atomic resolution were then fitted into these three density maps by rigid body fitting. The pre-60S (a) particle fills more completely the density map obtained by subtomogram averaging than the pre-40S particle (b). However, at this resolution, the pre-40S and pre-60S particles cannot be unambiguously distinguished from one another and display similar dimensions.

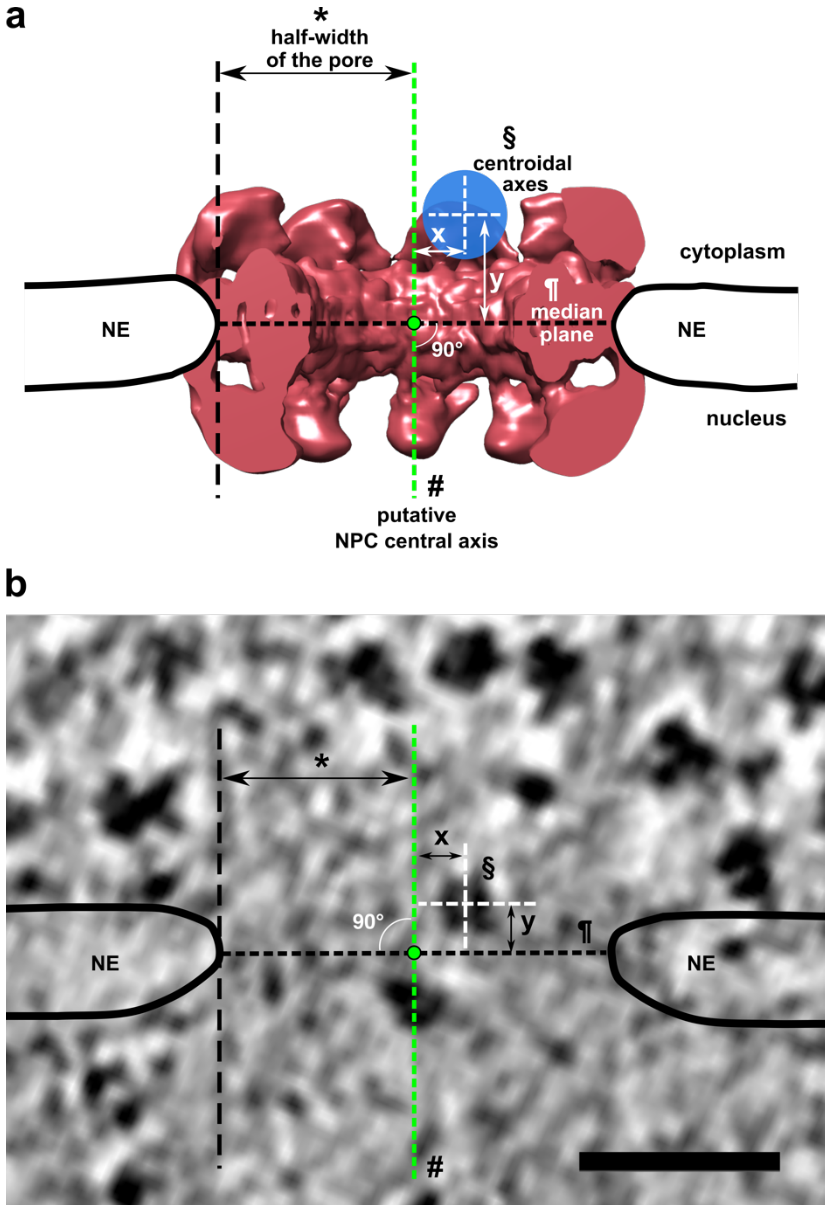

Supplementary Figure 2. Assignment of coordinates to pre-ribosomes relative to the central axis and the median plane of the NPC. (**a**) 3D view of the yeast NPC showing the coordinate system used to position pre-ribosomal particles (blue sphere) relative to the median plane (x axis; black dotted line) and the central axis (y axis; green dotted line) of the NPC. (**b**) Example of coordinate assignment to RNP particles on a tomographic section. This subtomogram shows two pre-ribosomal particles crossing a NPC. Scale bar: 50 nm.

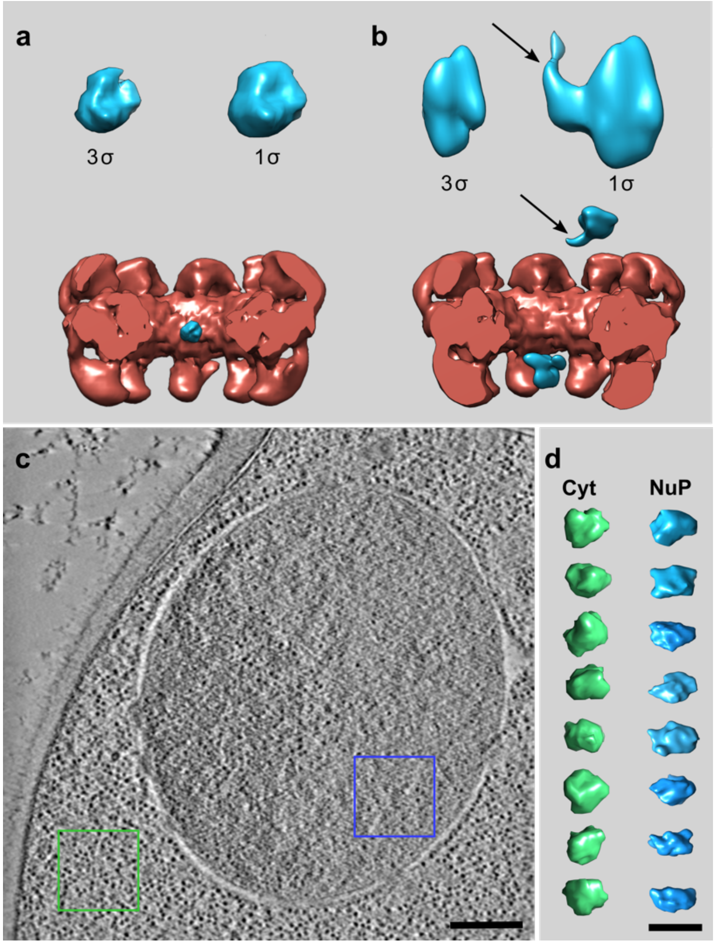

Supplementary Figure 3. Additional filamentous densities are detected on pre-ribosomes located on the cytoplasmic side of the NPC. (**a**) Such additional densities were not observed on pre-ribosomal particles located in the inner ring of the NPC, as shown here with two grey level cut-off (1σ or 3 σ; σ : standard deviation of the grey level distribution) for segmentation of the particles by isosurfacing. (**b**) Subtomogram showing two pre-ribosomal particles located in the same NPC; additional densities are only found on the particle located on the cytoplasmic side (black arrow). (**c**) Thin section of the tomogram performed on wild-type cells. Extraction of cytoplasmic ribosomes (blue square) and nucleoplasmic pre-ribosomes (green square) are shown in panel d. Scale bar: 500 nm. (**d**) Mature cytoplasmic ribosomes (green, Cyt) and nuclear particles (blue, NuP) do not display additional densities (grey level cut-off: 1.5 σ). Scale bar: 20 nm.
